# Corrigendum: Antagonistic Coevolution Limits the Range of Host Defense in *C. elegans* Populations

**DOI:** 10.3389/fcimb.2022.902233

**Published:** 2022-05-11

**Authors:** Jordan A. Lewis, McKenna J. Penley, Hannan Sylla, Sebastián Durán Ahumada, Levi T. Morran

**Affiliations:** ^1^ Department of Biology, Emory University, Atlanta, GA, United States; ^2^ Population Biology, Ecology, and Evolution Graduate Program, Emory University, Atlanta, GA, United States; ^3^ Department of Environmental Sciences, Emory University, Atlanta, GA, United States

**Keywords:** coevolution, experimental evolution, host defense, *Caenorhabditis elegans*, *Serratia marcescens*, antagonistic coevolution

## Text Correction

In the original article, there was an error. In the keywords both *Caenorhabditis elegans, Serratia marcescens* were misspelled as “Ceanorhabditis elegans” and “Serratia macrescens”.

A correction has been made to ***The Keywords***:

**Keywords: coevolution, experimental evolution, host defense, *Caenorhabditis elegans*, *Serratia marcescens*, antagonistic coevolution**

**Error in Figure/Table**

In the original article, there was a mistake in **Figure 2** and **Figure 3** as published. **The figures were of low pixel quality and were difficult to read.** The corrected **Figure 2** and **Figure 3** appears below.

**Figure 2:**
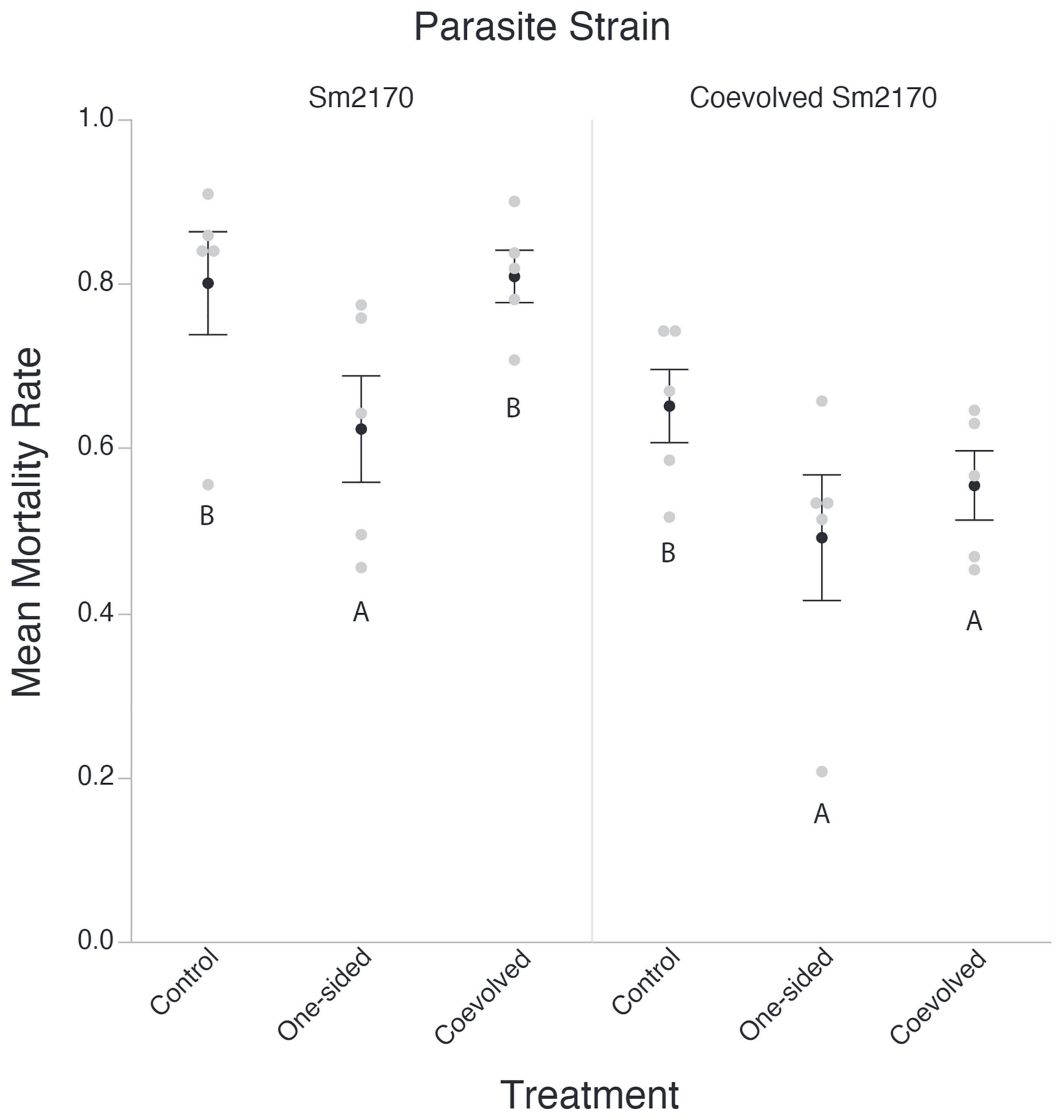
Host Mortality across related parasites. For each mortality assay 200 Worms were exposed to S. marcescens for a period of 48 hours using Serratia Selection Plates. Surviving worms were counted and the mortality is expressed as (worms plated – worms counted)/worms plated). Black circles represent the average mortality rate across all host populations for each bacterial treatment group. White circles represent the average mortality rate across all replicates for one host population. Points which share letters are statistically indistinguishable from each other, and only apply within their respective column. Error bars represent standard error. Letters are differentiated by a= 0.05.

**Figure 3:**
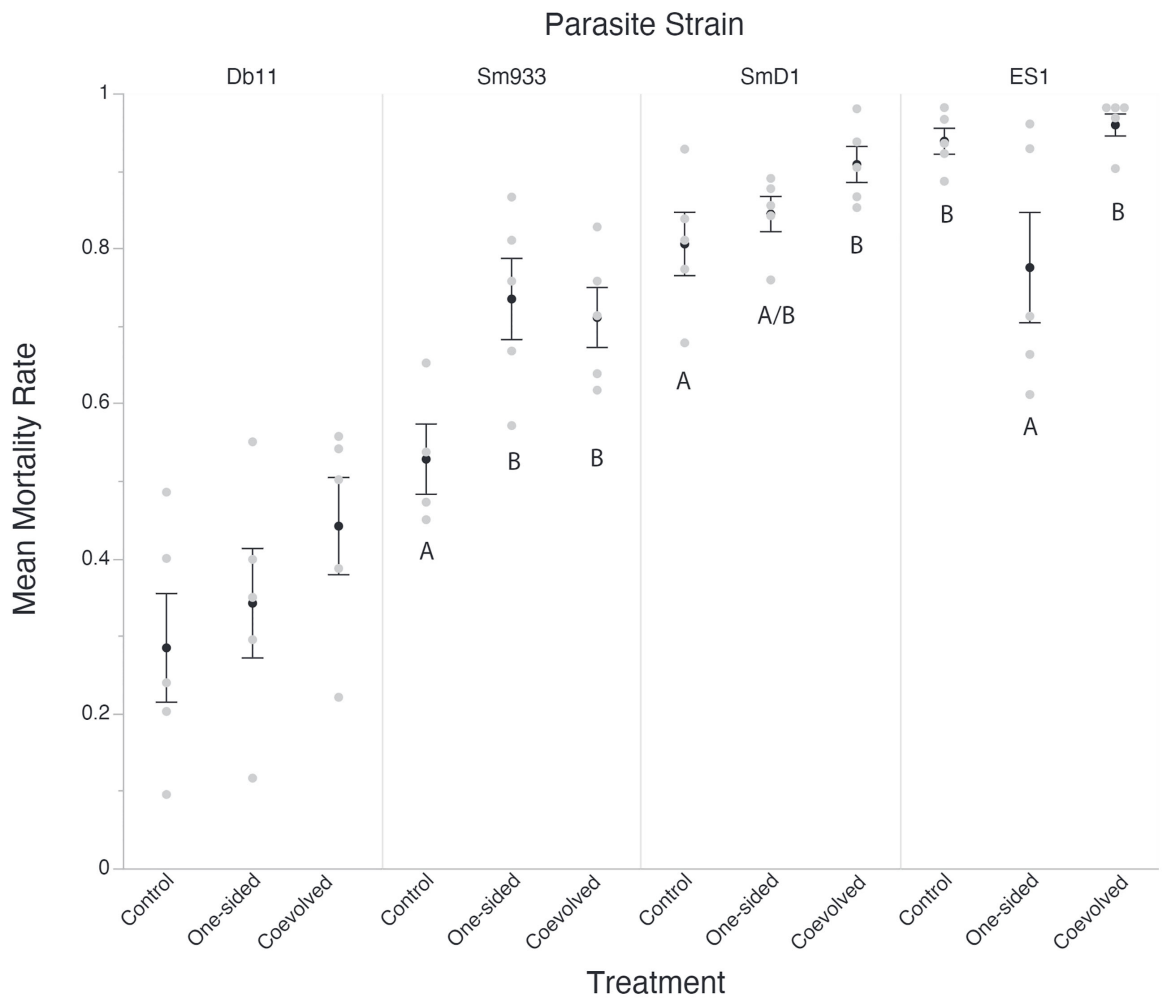
Host Mortality across unrelated parasites. For each mortality assay 200 Worms were exposed to S. marcescens for a period of 48 hours using Serratia Selection Plates. Surviving worms were counted and the mortality is expressed as (worms plated – worms counted)/worms plated). Black circles represent the average mortality rate across all host populations for each bacterial treatment group. White circles represent the average mortality rate across all replicates for one host population. Points which share letters are statistically indistinguishable from each other, and only apply within their respective column. Error bars represent standard error. Letters are differentiated by a= 0.05.

The authors apologize for this error and state that this does not change the scientific conclusions of the article in any way. The original article has been updated.

## Publisher’s Note

All claims expressed in this article are solely those of the authors and do not necessarily represent those of their affiliated organizations, or those of the publisher, the editors and the reviewers. Any product that may be evaluated in this article, or claim that may be made by its manufacturer, is not guaranteed or endorsed by the publisher.

